# Effects of Different Body Postures on Handgrip Strength Measurements Among Young Adults: A Preliminary Comparison of Standing, Chair-Seated, and Wheelchair-Seated Positions

**DOI:** 10.7759/cureus.95146

**Published:** 2025-10-22

**Authors:** Yurie Kawase, Saki Arai, Ikki Yoshida, Yohei Sawaya

**Affiliations:** 1 Department of Physical Therapy, School of Health Sciences, International University of Health and Welfare, Otawara, JPN; 2 Department of Rehabilitation, International University of Health and Welfare SHIOYA Hospital, Otawara, JPN

**Keywords:** grip strength, posture, sitting, standing, wheelchairs

## Abstract

Introduction: Although the influence of posture on handgrip strength measurement has been previously reported, no study has examined the wheelchair-seated position. This study aimed to compare handgrip strength values between the standing, chair-seated, and wheelchair-seated positions.

Materials and methods: This prospective observational study was conducted between May and June 2025 and included 29 university medical students from the International University of Health and Welfare in Otawara, Japan. Handgrip strength was measured under three conditions, i.e., standing, chair-seated, and wheelchair-seated, at one-week intervals. Statistical analyses were performed using one-way repeated-measures analysis of variance with Bonferroni post hoc comparisons.

Results: Handgrip strength values were 36.2 ± 10.2 kg in the standing position, 35.0 ± 10.0 kg in the chair-seated position, and 34.1 ± 9.6 kg in the wheelchair-seated position, showing a descending order from standing to chair-seated to wheelchair-seated. The handgrip strength in the wheelchair-seated position was significantly lower than that in the standing position. When the standing position was defined as 100%, the relative values were 97.0 ± 8.3% for chair-seated and 94.8 ± 8.0% for wheelchair-seated, with the handgrip in the wheelchair-seated significantly lower than that in the standing position.

Conclusions: Handgrip strength was influenced by body posture during measurement. To ensure accurate evaluation of longitudinal changes and intervention effects in the same individual, it is important to standardize the postural conditions during handgrip strength testing.

## Introduction

Handgrip strength is a key indicator of overall muscle strength. It is used worldwide because of its simplicity, high reproducibility, and strong prognostic value. Particularly in older adults, handgrip strength has been shown to correlate more strongly with total body muscle mass than with lower limb muscle strength [1], making it a common indicator of overall muscle strength. Moreover, low handgrip strength has been linked to negative outcomes, such as increased mortality risk, onset of cardiovascular disease, hospitalization, and cognitive decline [2-4]. Recently, its clinical significance as an integral part of the assessment of frailty and sarcopenia has grown [5,6].

Therefore, it is essential to establish an accurate method for measuring handgrip strength to properly assess and evaluate treatment efficacy. Factors influencing handgrip strength measurements include posture, number of measurements, fatigue, and the equipment used [6-8]. Among these, posture is particularly important. The Asian Working Group for Sarcopenia (AWGS) 2019 criteria for the diagnosis of sarcopenia recommend measuring handgrip strength in a standing position when using the Smedley dynamometer, which is widely used in Asia and Japan.

However, for older adults who are unable to maintain a standing position without assistance, seated measurements are considered appropriate [6]. While previous studies have compared the influence of posture on handgrip strength in standing and seated positions [8], to date, no study has explored the effects of wheelchair-seated posture. In Japan, the number of wheelchair users among older individuals in need of long-term care has steadily increased, reflecting the increasing demand for rehabilitation and caregiving in the wheelchair-seated position [9,10]. In rehabilitation settings within the long-term care insurance system, it is often difficult to measure physical conditions in standing or seated positions [11], and handgrip strength is sometimes measured while the individual is seated in a wheelchair. However, there is currently no evidence on how handgrip strength measurements in wheelchair-seated positions differ from those in other postures. We hypothesized that handgrip strength measured in the wheelchair-seated position would be lower than that measured in other postures. Clarifying these differences across postural conditions is important to enhance the accuracy of clinical assessments.

Therefore, this study aimed to investigate the effects of different measurement positions by measuring handgrip strength in university students under three conditions: standing, seated in a chair, and seated in a wheelchair. As a preliminary study in healthy young adults, the findings provide a fundamental step toward understanding how posture influences handgrip strength and may serve as a reference for future studies examining posture-related effects in older adults and individuals with limited mobility.

## Materials and methods

Research design

This prospective observational study was conducted from May to June 2025 and included 34 second- and third-year students from the International University of Health and Welfare in Otawara, Japan.

A priori sample size calculation

The required sample size was determined before the study. Assuming a one-way repeated-measures analysis of variance (ANOVA) with an effect size of 0.25, power of 0.8, three repeated measurements, and a significance level of 5%, the required sample size was determined to be 28 participants.

Research participants

**Figure 1:**
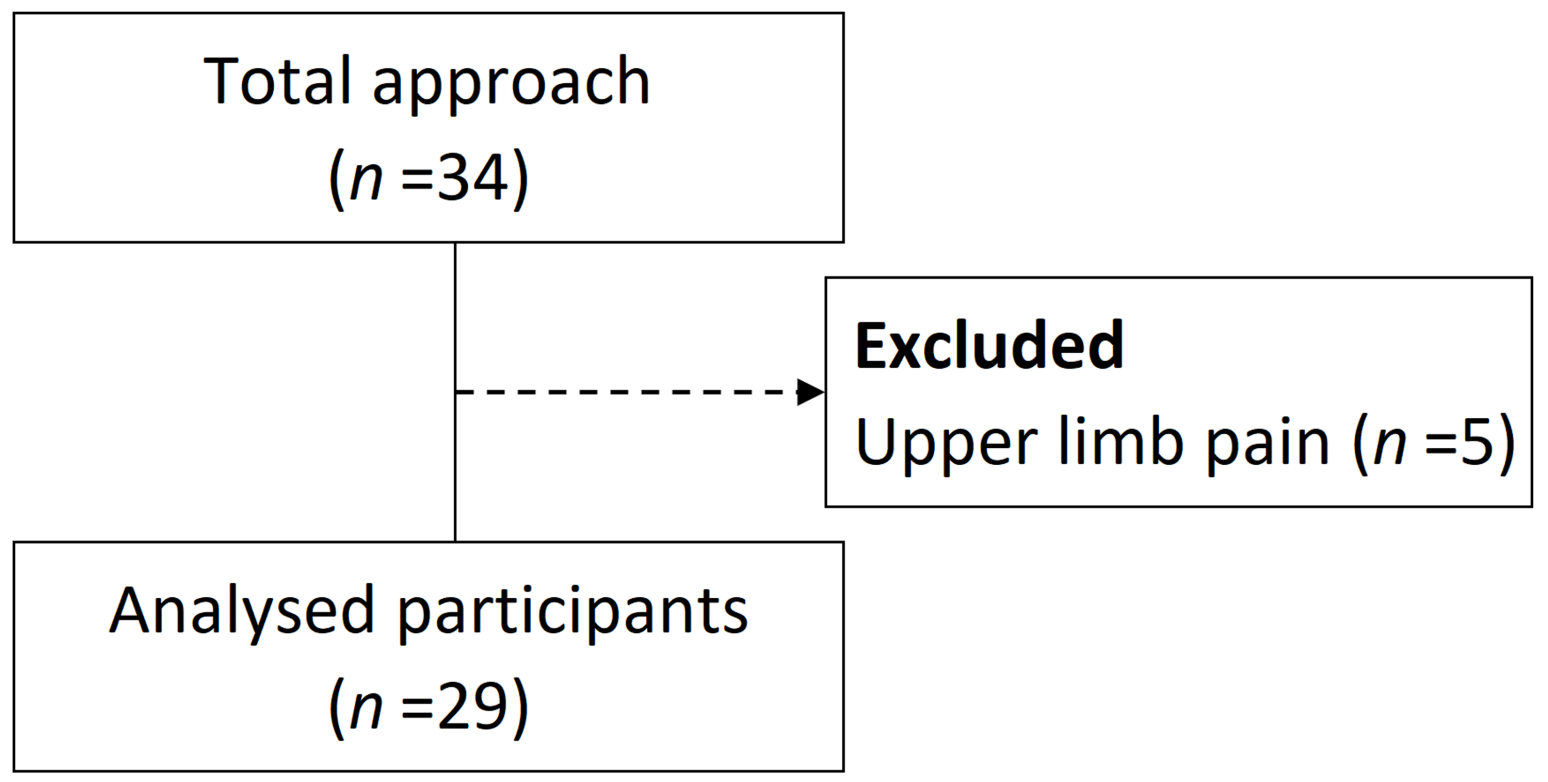
Participant flowchart

The participants were enrolled through verbal announcements. Anticipating a dropout rate of approximately 10%, 34 participants were initially recruited. Among them, five students who reported upper limb pain rated at ≥1 on the Numerical Rating Scale (NRS; 0 = no pain to 10 = worst pain) on the day of measurement were excluded. Consequently, 29 participants (16 males, 13 females; mean age, 19.8 ± 0.7 years) were included in the analysis (Figure 1).

Handgrip strength measurement

**Figure 2:**
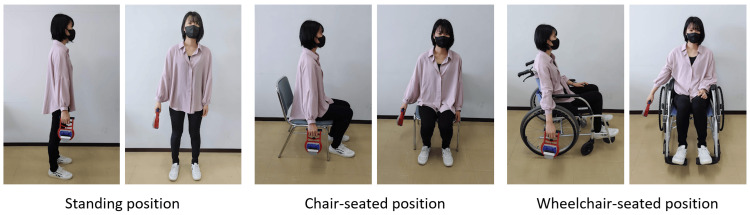
Handgrip strength measurement positions

Handgrip strength was measured under three conditions: standing, chair-seated, and wheelchair-seated. To calculate the minimum detectable change (MDC), standing measurements were performed twice as described in the statistical section. Accordingly, each participant completed one handgrip strength measurement session per week over four consecutive weeks, covering four conditions: standing (first), standing (second), chair-seated, and wheelchair-seated. To minimize order effects, the sequence of the four weekly sessions was randomized across participants. A Smedley-type digital grip dynamometer (TKK 5401 Grip-D; Takei Scientific Instruments, Niigata, Japan) was used for all the measurements. The handle was adjusted such that the proximal interphalangeal joint of the index finger was flexed at 90°, with the wrist in a neutral position. The numerical settings of the handle were recorded and consistently maintained for each participant throughout all sessions. For all postures (standing, chair-seated, and wheelchair-seated), the participants were instructed to keep their feet shoulder-width apart, with their arms held approximately 5 cm away from the trunk and elbows fully extended during testing (Figure 2). In the wheelchair-seated position, the participants placed their feet on footrests, naturally leaned slightly against the backrest, avoided contact with the arm supports and wheels by keeping their arms approximately 5 cm away, and maintained their shoulders in slight abduction. According to the AWGS 2019 protocol, two trials were performed on each side under each condition [6]. Among the four trials, the maximum value, irrespective of hand dominance, was used for analysis [6]. To minimize fatigue, approximately 60 seconds of rest was provided between trials [7]. The handgrip strength values were not disclosed to the participants, and verbal instructions during the measurements were standardized.

Wheelchair

A standard wheelchair defined by the Japanese Industrial Standards (JIS T 9201:2006) was used in this study (ECO-201B; MATSUNAGA, Gifu, Japan). The back support was set at a seat-back angle (θ_SB) of 100°, which corresponds to the standard specification. The footrest height was set at the standard value of approximately 50 mm from the floor. The specifications of the wheelchair were as follows: caster, six inches; rear wheel, 22 inches; front seat height, 430 mm; rear seat height, 400 mm; total length, 955 mm; total height, 885 mm; total width, 665 mm; and weight, 12.9 kg.

Statistics

Repeated-measures one-way ANOVA, followed by Bonferroni post-hoc tests, were then applied to compare handgrip strength measurements taken in the standing position (day 1), chair sitting position, and wheelchair sitting position. In addition, handgrip strength values for the seated and wheelchair-seated positions were expressed as percentages (with standing (day 1) values set at 100%) and compared using repeated-measures ANOVA with Bonferroni correction for multiple comparisons, in the same manner as the raw values.

In addition, the MDC for handgrip strength in university students was calculated from standing measurements obtained on days one and two. To calculate the MDC, we first examined the presence of fixed and proportional biases. Fixed bias was assessed using equation (i): if the 95% confidence interval, derived from the mean (mean) and standard deviation (SD) of the paired differences, did not include zero, fixed bias was considered present; otherwise, it was considered absent [12].

(i) Mean ± t(n−1) × SD / √​​n [12]

t(n−1): t-distribution with n−1 degrees of freedom

Proportional bias was considered present when the slope of the regression equation for the mean and the difference between the two measurements were statistically significant [13]. The MDC and the standard error of measurement (SEM) were then calculated using the following equation (ii).

(ii) MDC = 1.96 × √2 × SEM [14,15]

SEM = SD / √2 [16]

All statistical analyses were performed using IBM SPSS Statistics for Windows, Version 26.0 (released 2018, IBM Corp., Armonk, NY) and G*Power version 3.1.9.2 (Heinrich Heine University Düsseldorf, Germany), with the significance level set at 5%.

## Results

Table 1 summarizes the baseline characteristics of the participants.

**Table 1:** Participants' baseline characteristics Values are presented as numbers or mean ± standard deviation.

Assessment items	Data
Age (year)	19.8 ± 0.7
Male/Female (number)	16/13
Height (cm)	167.2 ± 8.4
Weight (kg)	60.1 ± 10.7
Body mass index (kg/m^2^)	21.4 ± 2.6

**Figure 3:**
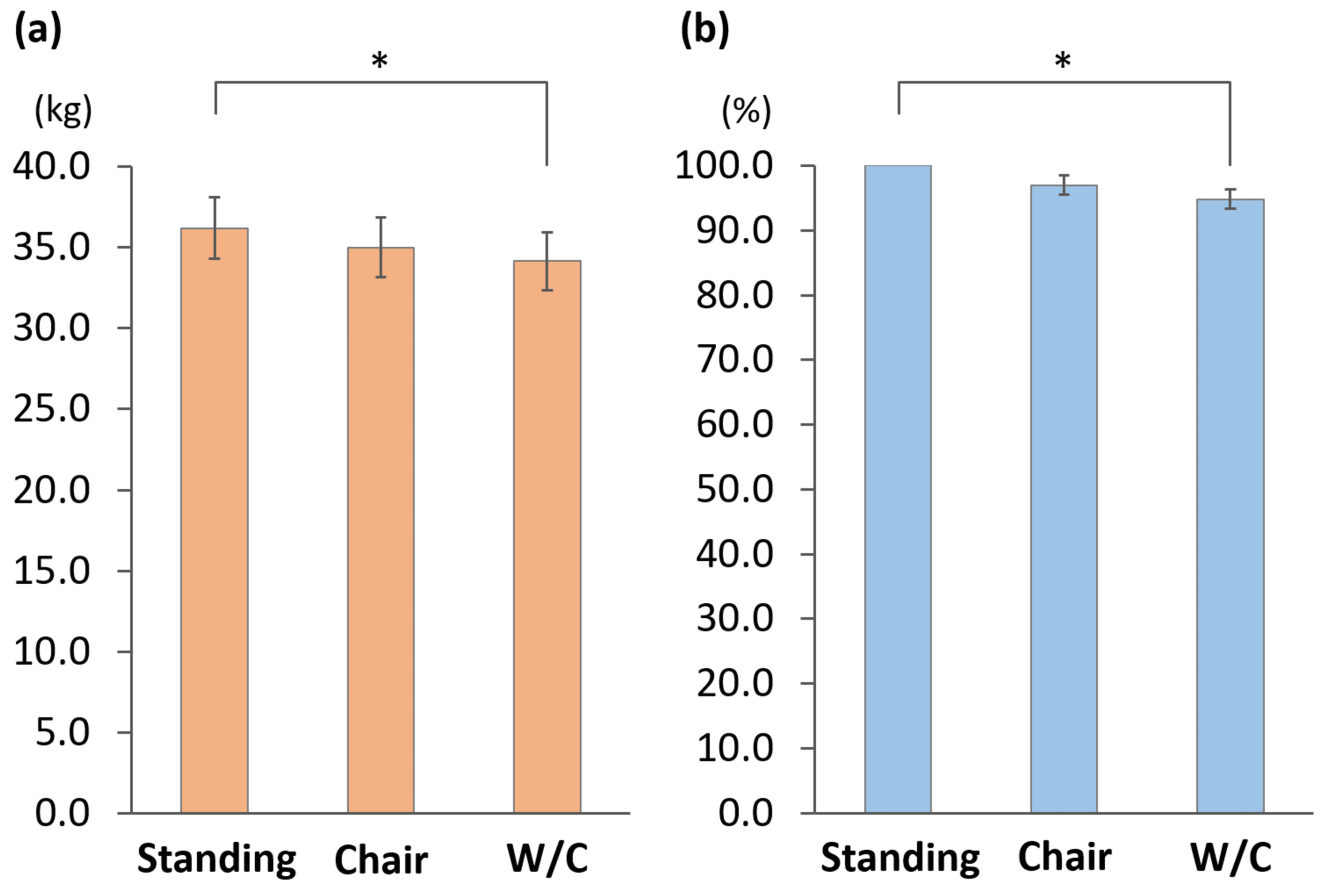
Results of handgrip strength in standing, chair-seated, and wheelchair-seated positions (a) Raw values and (b) percentages relative to standing, defined as 100%. (a) Repeated-measures analysis of variance (ANOVA) revealed a significant difference among the positions (P = 0.001), and Bonferroni post-hoc analysis indicated that grip strength in the wheelchair-seated position was significantly lower than that in the standing position (P = 0.004). (b) Repeated-measures ANOVA revealed a significant difference (P = 0.003), and post-hoc comparisons showed that the percentage in the wheelchair-seated position was significantly lower than that in the standing position (P = 0.004). The error bars indicate the standard error. W/C: wheelchair. *: p < 0.05

Figure 2 shows the handgrip strength results under the three conditions: standing, chair-seated, and wheelchair-seated. The mean values were 36.2 ± 10.2 kg in the standing position, 35.0 ± 10.0 kg in the chair-seated position, and 34.1 ± 9.6 kg in the wheelchair-seated position, showing a progressive decrease from standing to chair-seated to wheelchair-seated. Repeated-measures ANOVA revealed a significant difference between the positions (P = 0.001), and Bonferroni post-hoc analysis indicated that handgrip strength in the wheelchair-seated position was significantly lower than that in the standing position (P = 0.004) (Figure 3a). When the first standing measurement was defined as 100%, the relative values were 97.0 ± 8.3% for the chair-seated position and 94.8 ± 8.0% for the wheelchair-seated position. Analysis of these relative values also demonstrated a significant difference by repeated-measures ANOVA (P = 0.003), with Bonferroni post hoc comparisons showing that the wheelchair-seated position was significantly lower than those in the standing position (P = 0.004) (Figure 3b).

Systematic error analysis using standing measurements on days one and two revealed no fixed or proportional bias. The MDC was 4.4 kg, and the %MDC was 12.3% (Table 2).

**Table 2:** Minimal detectable changes in grip strength among university students The MDC was calculated from the standing measurements obtained on days 1 and 2. Differences were calculated as day 2 minus day 1. CI: confidence interval; MDC: minimal detectable change; SEM: standard error of measurement

Assessment items	Data
Measurement values (kg)	
Day 1	36.2 ± 10.2
Day 2	35.4 ± 9.5
Average	35.8 ± 9.8
Difference	-0.8 ± 2.2
Fixed bias	Non-existent
95% CI	-1.60~0.10
Proportional bias	Non-existent
Slope	-0.075
P value	0.081
SEM	1.6
MDC	4.4
%MDC	12.3%

## Discussion

This study is the first to investigate how wheelchair-seated positions affect handgrip strength compared with standing and chair-seated positions. The analysis showed that the handgrip strength decreased in the following order: standing > chair seated > wheelchair seated. Although no significant difference was observed between the standing and chair-seated positions, handgrip strength in the wheelchair-seated position was 5.2% lower than that in the standing position. These findings suggest that a wheelchair-seated posture may restrict the maximal expression of handgrip strength. As measuring handgrip strength in the standing position is often challenging for older individuals requiring high levels of care, assessments are frequently performed in the wheelchair-seated position. Therefore, understanding how posture affects handgrip strength and standardizing the testing posture, particularly in longitudinal clinical and research settings involving older adults who use wheelchairs, is essential to ensure accurate, reliable handgrip assessment.

Regarding the influence of posture on handgrip strength measurement, El-Sais et al. investigated handgrip strength in the supine, lateral, prone, sitting, and standing positions. Handgrip strength was highest in the standing position and significantly lower in the prone position than in both the standing and sitting positions, whereas no significant differences were observed among the supine, lateral, and prone positions [17]. Furthermore, Balogun et al. compared the handgrip strength between standing and sitting positions and reported significantly greater values in the standing position [18]. Several other studies have also reported that handgrip strength is higher in the standing position [19-22]. Physiologically, this may be explained by the increased temporal and spatial summation of contracting muscles in the standing position, where postural maintenance activates the trunk and lower limb muscles, thereby elevating the overall muscle activity. As a result, neural stimuli are delivered to muscle fibers at a higher frequency (temporal summation) and a greater number of muscle fibers are recruited simultaneously (spatial summation), which may facilitate greater handgrip strength [17]. Moreover, when participants adopt a standing posture, it is believed that their optimal mental and physical condition is most effectively reproduced [23]. In particular, standing enhances the synergistic activity between the upper- and lower-limb muscles, allowing participants to generate greater handgrip strength. Furthermore, previous studies have reported that cortical activity and peripheral arousal are more effectively facilitated in the standing position [18,24], which likely contributes to the increased handgrip strength observed. By contrast, in the sitting position, sensory feedback from the muscles and joints of the lower limbs to the brain is markedly reduced [19], making it difficult to achieve the same synergistic effects and arousal facilitation as in standing.

These physiological factors may explain why no significant difference in handgrip strength was observed between standing and chair-seated positions, whereas a significant reduction was evident only in the wheelchair-seated position. In wheelchair sitting, the lower limb and trunk muscle tension tends to decrease more readily than in chair sitting, and is influenced by factors such as foot placement on the footrests and contact between the body, backrest, and side guards. Leaning against the backrest of the wheelchair likely induces a posterior pelvic tilt, which may reduce muscle activation and grip strength. Furthermore, maintaining the shoulder joint in slight abduction while sitting in a wheelchair may reduce upper limb stability and impair the contraction efficiency of the forearm flexor muscles, thereby limiting the maximal handgrip strength.

The results of this study demonstrated that handgrip strength is influenced by the posture adopted during measurement, which should be carefully considered when evaluating handgrip strength in older individuals who use wheelchairs. As handgrip strength tends to be lower in the wheelchair-seated position than in the standing or chair-seated positions, direct comparisons of measurements obtained in different postures may lead to misinterpretations. Posture standardization during measurement is essential to accurately assess longitudinal changes or intervention effects in the same individual. Although the difference in handgrip strength between the standing and wheelchair-seated positions was statistically significant, it remained within the MDC range, suggesting that the effect of posture may fall within the measurement error. Moreover, since the standard deviations were large and the standing and wheelchair-seated grip strengths might overlap, the clinical significance of this difference may be somewhat limited.

This study has several limitations. First, as this preliminary investigation was conducted exclusively in healthy young adults, the extent to which posture influences handgrip strength in older adults or individuals requiring nursing care remains uncertain, underscoring the need for further studies in these populations. Second, only one type of wheelchair was used; therefore, the extent of side-guard contact varied according to the participants’ body size, potentially limiting the generalizability of the findings to other wheelchair models with different specifications. Nevertheless, the wheelchair employed in this study was defined by the Japanese Industrial Standards and can be regarded as a widely used representative model. In addition, to avoid contact with the arm supports and rear wheels, the shoulder abduction angle becomes naturally slightly larger in the wheelchair-seated position than in other postures. Third, objective assessments such as electromyography were not performed to evaluate trunk and lower limb muscle activities; therefore, a causal link between posture-related changes in muscle activity and reduced handgrip strength could not be established. Fourth, to minimize variations in handgrip strength due to temporal factors or the occurrence of any events associated with an extended measurement period, the study period was set to approximately one month, and the MDC was calculated only in the most standard standing posture.

As a preliminary study conducted in healthy young adults, these findings provide a foundational step toward understanding how posture may influence handgrip strength; however, it remains uncertain whether the results obtained from university students can be directly applied to older adults. Therefore, we once again emphasize the importance of conducting further studies with a similar design in older adults and wheelchair users to verify these findings and clarify their clinical significance.

## Conclusions

This study examined the effects of different measurement positions on handgrip strength. Handgrip strength decreased in the order: standing, chair sitting, and wheelchair sitting, with an effect particularly evident in wheelchair sitting, where values were approximately 5.2% lower than those of standing. Establishing standardized measurement conditions is essential to improve the reliability of longitudinal evaluations and the comparability of intervention studies.
